# *In silico *analysis of HLA associations with drug-induced liver injury: use of a HLA-genotyped DNA archive from healthy volunteers

**DOI:** 10.1186/gm350

**Published:** 2012-06-25

**Authors:** Ana Alfirevic, Faviel Gonzalez-Galarza, Catherine Bell, Klara Martinsson, Vivien Platt, Giovanna Bretland, Jane Evely, Maike Lichtenfels, Karin Cederbrant, Neil French, Dean Naisbitt, B Kevin Park, Andrew R Jones, Munir Pirmohamed

**Affiliations:** 1 Department of Molecular and Clinical Pharmacology, Institute of Translational Medicine, University of Liverpool, The Waterhouse Building, Brownlow Street 1-5, Liverpool, L69 3GL, UK; 2 Department of Functional and Comparative Genomics, Institute of Integrative Biology, University of Liverpool, Biosciences Building, Crown Street, Liverpool, L69 7ZB, UK; 3 Department of Molecular and Clinical Pharmacology, Institute of Translational Medicine, University of Liverpool, The Sherrington Building, Ashton Street, Liverpool, L69 3GE, UK; 4 Safety Assessment, AstraZeneca, Gartuna, Södertälje, Sweden

## Abstract

**Background:**

Drug-induced liver injury (DILI) is one of the most common adverse reactions leading to product withdrawal post-marketing. Recently, genome-wide association studies have identified a number of human leukocyte antigen (HLA) alleles associated with DILI; however, the cellular and chemical mechanisms are not fully understood.

**Methods:**

To study these mechanisms, we established an HLA-typed cell archive from 400 healthy volunteers. In addition, we utilized HLA genotype data from more than four million individuals from publicly accessible repositories such as the Allele Frequency Net Database, Major Histocompatibility Complex Database and Immune Epitope Database to study the HLA alleles associated with DILI. We utilized novel *in silico *strategies to examine HLA haplotype relationships among the alleles associated with DILI by using bioinformatics tools such as NetMHCpan, PyPop, GraphViz, PHYLIP and TreeView.

**Results:**

We demonstrated that many of the alleles that have been associated with liver injury induced by structurally diverse drugs (flucloxacillin, co-amoxiclav, ximelagatran, lapatinib, lumiracoxib) reside on common HLA haplotypes, which were present in populations of diverse ethnicity.

**Conclusions:**

Our bioinformatic analysis indicates that there may be a connection between the different HLA alleles associated with DILI caused by therapeutically and structurally different drugs, possibly through peptide binding of one of the HLA alleles that defines the causal haplotype. Further functional work, together with next-generation sequencing techniques, will be needed to define the causal alleles associated with DILI.

## Background

Drug-induced T-cell mediated hypersensitivity reactions are feared by clinicians and pharmaceutical companies alike. Although these reactions occur infrequently, they are still a cause of severe morbidity and mortality. They are difficult to predict during drug discovery because of a lack of *in vitro *or animal models and, because they occur at such a low frequency, they are often only detected during the late stages of drug development or, more commonly, during post-marketing. Cutaneous rashes are the most common manifestations and may be accompanied by systemic symptoms such as fever. In addition, extracutaneous organ involvement, such as drug-induced liver injury (DILI) can also occur, either in combination with skin involvement or in isolation. Taken together, the occurrence of immune-mediated drug hypersensitivity poses a challenge with respect to prediction, diagnosis and therapy.

A number of hypotheses have been proposed to explain the ability of drugs to initiate an immune response against host cells. Drugs or drug metabolites may bind covalently to proteins before being taken up by specialized antigen-presenting cells [1]. These haptenated proteins are then processed within the cell, and cleaved into peptide fragments that can be presented to T cells via major histocompatibility complex (MHC) class I or II molecules [2]. Alternatively, the 'pharmacological interaction with immune receptors' (p-i) concept suggests that the interaction between drug, T-cell receptor and MHC molecule can be non-covalent and that direct stimulation of T cells can occur, independent of cellular processing [3]. Both of these mechanisms highlight that the unique interaction between drug, T-cell receptor and MHC molecule is a key factor in the development of immune-mediated adverse reactions to drugs and, as such, the study of HLA alleles represents a logical route to study the genetic basis of such immune-mediated reactions.

Consistent with this, several genome-wide association studies conducted recently have identified a number of HLA alleles that are associated with a range of clinically diverse hypersensitivity reactions to structurally unrelated low-molecular weight compounds (Table 1) [4-22]. HLA alleles reside in the MHC region on chromosome 6, the most polymorphic region in the human genome [23]. Strong linkage disequilibrium (LD) extends across the whole MHC and includes the human leukocyte antigen (HLA) class I, class II and class III genes [24]. Given the extensive LD, it is difficult to prove that any reported associations with HLA alleles are causal. The crucial role of HLA molecules in immune recognition and evidence for the involvement of drug-specific T cells in several of these HLA-associated reactions indicates that the immune system is involved but does not implicate a particular HLA allele [25-28]. A functional role for HLA class I alleles has only been demonstrated for abacavir-induced hypersensitivity [29]. In addition, potential clinical utility of HLA genetic markers has been demonstrated recently for efficacy and risk management of patients treated with interferon-beta for multiple sclerosis [30,31].

**Table 1:** HLA alleles associated with adverse drug reactions and cell availability in the HLA-typed archive

					Number of carriers of HLA alleles associated with DILI in our cohort (*n *= 385)	
					
Drug	HLA allele	Reaction (ethnicity)	Odds ratio (95% CI)	Reference	Hmz	Htz
**Drug-induced hypersensitivity reactions**						
Abacavir	B*57:01	Hypersensitivity (all)	117 (29-481)	[14]	1	25
Carbamazepine	B*15:02	SJS/TEN (Han Chinese)	2,504 (126-49,522)	[4]	1	6
	A*31:01	All phenotypes (Caucasian)	12.12 (4.03-20.65)	[17]	0	23
Allopurinol	B*58:01	SJS (Han Chinese)	580.3 (34.4-9,780.9)		0	6
		SJS (Caucasian)	80 (34-187)	[10,12]		
Nevirapine	DRB1*01:01	Hypersensitivity (Caucasian)	4.8 (1.3-16.8)	[16]	3	52
Lamotrigine	B*38	SJS/TEN (Caucasian)	6.8 (2.6-18)	[12]	0	7
						
**Drug-induced liver injury**						
Flucloxacillin	B*57:01	DILI (Caucasian)	80.6 (22.8-284.9)	[6]	1	25
Ximelagatran	DRB1*07:01	DILI (Caucasian)	4.4 (2.2-8.9)	[11]	10	91
	DQA1*02:01		4.4 (2.2-8.1)		ND	ND
Lumiracoxib	DRB1*15:01	DILI (Caucasian)	7.5 (5.0 to 11.3)	[21]	10	100
	DQA1*01:02		6.3 (4.1 to 9.6)		ND	ND
	DQB1*06:02		6.9 (4.6 to 10.3)		8	90
Co-amoxiclav	DRB1*15:01 and	DILI (Caucasian)	2.8 (2.1 to 3.8)	[7,8,13,18]	10	100
	DQB1*06:02				8	90
	A*02:01		2.3 (1.8 to 2.9)		46	153
Antituberculosis (isoniazid, rifampicin, pyrazinamide)	DQB1*02:01	DILI (Indian)	1.9 (1.0 to 3.9)	[20]	20	143
Lapatinib	DQA1*02:01	DILI (Caucasian)	2.2 (1.1 to 5.7)	[22]	ND	ND
			9.0 (3.2 to 27.4)			
	DRB1*07:01		6.9 (2.5 to 19.9)		10	91
	DQB1*02:02		6.9 (2.5 to 19.6)		0	0
Diclofenac	DRB1*13	DILI (Caucasian)	-	[5]	-	-
Clometacin	B*08	DILI	-	[19]	-	-
Ticlopidine	A*33:03	DILI (Japanese)	13.0 (4.4 to 38.6)	[9]	1	8

Human leukocyte antigen (HLA) alleles associated with hypersensitivity reactions and drug-induced liver injury (DILI) are presented. All but two DILI studies (one Japanese and one Indian) were conducted in individuals of Caucasian ancestry. The number of individuals whose peripheral mononuclear cells are stored in the HLA-typed archive (*n *= 385) and who are carriers of HLA alleles implicated in these reactions is shown. CI, confidence Interval; Hmz, homozygotes; Htz, heterozygotes; ND, not determined (DQA1 locus not genotyped in ENW cohort); SJS, Stevens Johnson syndrome; TEN, toxic epidermal necrolysis.

**Figure 1:**
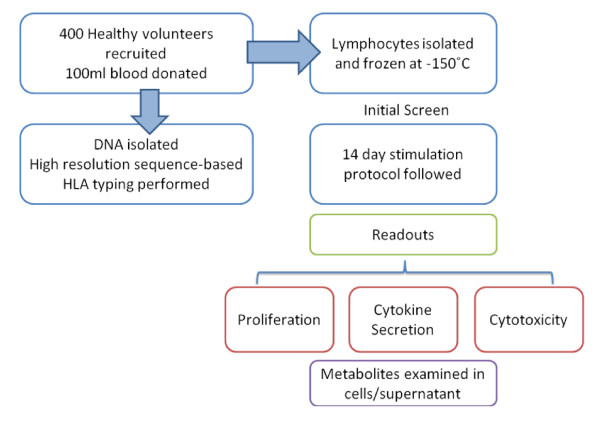
**Outline of the intended experimental strategy utilizing lymphocytes from HLA-genotyped volunteers**.

In order to study HLA-linked reactions *in vitro*, work with genetically characterized cells is essential. For example, immune cells containing specific HLA types can be utilized in functional assays to determine the propensity of small molecules such as drugs to initiate T-cell responses (Figure 1). This approach has already shown some success: drug-specific secretion of interferon-gamma was detected in lymphocytes isolated from HLA-B*57:01-positive abacavir-naïve healthy donors following a short priming period [29]. Bioinformatic approaches are also important to define the mechanisms of immune reactions that are associated with specific HLA types - this is now possible given that sequence-based HLA typing has become faster and cheaper, which has resulted in increasing availability of individual and frequency data in public repositories.

In this study, we demonstrate how an HLA-typed cell archive from 400 healthy volunteers combined with the use of bioinformatic tools to analyze publicly accessible information on more than four million HLA-typed individuals was utilized for the investigation of immune-mediated adverse drug reactions (ADRs). Specifically, we have used the alleles reported to predispose to DILI as a paradigm for our bioinformatic based approach.

## Materials and methods

### Volunteer recruitment and eligibility

The study was approved by the Liverpool Research Ethics Committee and all participants gave written informed consent. Advertisements were placed in local newspapers, on the University of Liverpool website and the Royal Liverpool University Hospital Bulletin. Volunteers were eligible to take part in the study if they were aged between 18 and 60 years, healthy and willing to donate one or more blood samples. The following exclusion criteria were applied and volunteers were not recruited if: they donated blood to transfusion services in the last 4 months; they had any medical problems, including asthma, diabetes, epilepsy or anemia; on any medications or if they had taken any recreational drugs in the last 6 weeks (including cannabis, speed, ecstasy, cocaine, LSD, and so on). Women were excluded if pregnant.

Healthy volunteers (*n *= 400) were recruited from North West England. Blood samples were taken from 385 individuals at the Royal Liverpool and Broadgreen University Hospital Clinical Research Facility [32]. Fifteen individuals were excluded because of difficult peripheral vein access or consent withdrawal. Confidentiality was maintained throughout the study by several mechanisms: first, only clinically trained personnel had access to participant's personal data, which are stored in password-protected computer files; second, coding of DNA samples and cells used in functional assays was performed; third, subjects were identified only by their assigned number and laboratory personnel were not aware of an individual's HLA status when performing immunological experiments. Participants were given the right to withdraw from further taking part in the study at any time. In that case, any identifiable data or tissue would be anonymized and retained or otherwise disposed of if specified by the participant.

A total of 100 ml of blood was collected for both DNA and peripheral blood mononuclear cell isolation. Careful consideration was paid to ensure that the amount of blood collected would enable the intended experimental strategy without asking too much from volunteers. All samples were processed within 4 hours of collection.

A total of 385 healthy unrelated individuals were included in the investigation of associations among DILI and HLA alleles. Of those, 298 individuals with Caucasian origin (77% of the study; Table S1a in Additional file 1) were analyzed to examine genetic traits and associations for these alleles.

This research has been conducted in accordance with the principles of the Declaration of Helsinki.

### Isolation of genomic DNA and sequence-based HLA typing

Genomic DNA was extracted from 10 ml venous blood using Chemagic magnetic separation (Chemagen, Baesweiler, Germany). DNA concentration was determined using the Nanodrop spectrophotometer (Labtech, East Sussex, UK) and picogreen measurements. High-resolution sequence-based HLA typing was performed by Histogenetics laboratory (Histogenetics, New York, NY, USA) at the following loci: HLA-A, -B, -C, -DRB1 and -DQB1. All allele names were validated against the International Immunogenetics project (IMGT)/HLA database release 3.3.0. We use a suffix 'g' to describe ambiguities (Table S5 in Additional file 1). Long-term storage of DNA is provided by SmaRTStore (Brooks Automation Limited, Brooks Life Science Systems, Irlam, Manchester, UK) a DNA archive with integrated robotics. Samples can be tracked and monitored by an electronic laboratory management system provided by STARLIMS. No clinical information on volunteers is available to laboratory personnel.

### Extraction of data from public databases

#### Allele Frequency Net Database

We used frequency data from the Allele Frequency Net Database (AFND) [33], which is a public repository for the dissemination of HLA allele frequencies in worldwide populations. Our analysis included HLA data from more than three million individuals containing haplotype frequency data, of which more than two million subjects were of Caucasian origin (Table S1b in Additional file 1). HLA allele frequencies for Caucasian individuals from this study (*n *= 298) were added to the AFND and coded as England North West (ENW). Allele and haplotype frequencies are available at the AFND website [33,34].

#### dbMHC database

We extended our analyses to datasets available on the Major Histocompatibility Complex database (dbMHC) [35], a public repository containing data previously submitted for the 13th International Histocompatibility Workshop for anthropological analysis [36]. We applied filtering by extracting only genotypes that were uniformly typed for all loci in a given population (Table S1c in Additional file 1). A total of 8,569 individuals that matched the criteria were selected from 77 populations.

### Sample size calculations and statistical methods

Sample size estimates for recruitment of volunteers were based on the frequency of the HLA-DRB1*07:01 allele because of our interest in ximelagatran-induced DILI. Allelic frequency for DRB1*07:01 varies from 0.087 in Swedish to 0.13 in Turkish and 0.19 in Dutch Caucasian populations [33]. From a previous study we conducted in Caucasian individuals from North West England where the frequency of DRB1*07:01 was 0.14 and assuming the Hardy-Weinberg distribution of genotypes, we estimated that, in a cohort of 400 individuals, we would identify more than 60 individuals who carry one DRB1*07:01 allele and approximately three DRB1*07:01 homozygous individuals.

Bioinformatics tools and resources used in the analysis of datasets are listed in Table 2.

**Table 2:** *In silico *analysis - bioinformatic tools and databases

Tool	URL	Description
NetMHCpan	http://www.cbs.dtu.dk/services/NetMHCpan/	A server for predicting the binding of peptide sequences to MHC molecules (class I) for humans and other species
NetMHCIIpan	http://www.cbs.dtu.dk/services/NetMHCIIpan/	A server for predicting the binding of peptide sequences to MHC molecules (class II) for humans and other species
Immune Epitope Database (IEDB)	http://www.immuneepitope.org/	A database storing data related to antibody and T-cell epitopes for humans, non-human primates, rodents, and other animal species
Allele Frequency Net Database (AFND)	http://www.allelefrequencies.net/	A database storing allele, haplotype and genotype frequencies in worldwide populations, sourced from over 1,200 studies, concerning immune-related genes (HLA, KIR, MIC and cytokine gene polymorphisms)
PyPop	http://www.pypop.org/	Open-source software package for the analysis of populations at large scale and multiple loci
GraphViz	http://www.graphviz.org/	Open source graph visualization software
dbMHC	http://www.ncbi.nlm.nih.gov/gv/mhc/main.cgi?cmd=init	A database providing DNA, protein and clinical data related to the human major histocompatibility complex (MHC)
PHYLIP	http://evolution.genetics.washington.edu/phylip.html	Open source software for inferring phylogenies
TreeView	http://taxonomy.zoology.gla.ac.uk/rod/treeview.html	Open source tree viewing software

KIR, Killer-cell immunoglobulin-like receptors; MIC, MHC class I chain related (MIC) genes.

### Allele and genotype frequencies

Allele frequencies were calculated by direct counting using the PyPop software [37]. To calculate genotype frequencies (percentage of individuals), we used an in-house application (AFND Frequency Calculator). Results are shown in Table 3. To analyze the world distribution of selected alleles associated with DILI, we compared their frequencies with global frequencies in populations submitted to AFND grouped by ethnicity (Table S4a in Additional file 1) or geographic region (Table S4b in Additional file 1). In addition, the HLA frequencies were also checked against dbMHC for calculation of quality control (Table S4c in Additional file 1).

**Table 3:** Allele and genotype frequencies in Caucasians from the HLA-typed archive described in the present study (North West England, *n *= 298)

HLA-A			HLA-B			HLA-C			HLA-DRB1			HLA-DQB1		
				
Allele	AF	GF (%)	Allele	AF	GF (%)	Allele	AF	GF (%)	Allele	AF	GF (%)	Allele	AF	GF (%)
A*01:01g	0.208	38.6	B*07:02g	0.153	27.5	C*01:02g	0.040	8.1	DRB1*01:01:01	0.086	16.1	DQB1*02:01g	0.273	48.0
A*01:06	0.002	0.3	B*07:05g	0.003	0.7	C*02:02:02	0.035	7.0	DRB1*01:02:01	0.007	1.3	DQB1*03:01g	0.154	28.2
A*02:01g	0.289	50.7	B*08:**01g**	0.154	29.9	C*03:02	0.003	0.7	DRB1*01:03	0.018	3.7	DQB1*03:02:01	0.116	21.5
A*02:05g	0.010	2.0	B*13:02	0.007	1.3	C*03:03g	0.064	12.8	DRB1*03:01:01	0.154	28.9	DQB1*03:03:02	0.049	9.7
A*02:20:01	0.002	0.3	B*14:01	0.010	2.0	C*03:04	0.076	14.4	DRB1*04:01:01	0.116	21.5	DQB1*03:04	0.003	0.7
A*02:24	0.002	0.3	B*14:02:01	0.025	5.0	C*04:01g	0.062	11.4	DRB1*04:02	0.008	1.7	DQB1*04:02	0.015	3.0
A*03:01g	0.138	25.5	B*15:01g	0.059	11.7	C*05:01g	0.102	19.5	DRB1*04:03:01	0.008	1.7	DQB1*05:01:01	0.112	20.5
A*03:02	0.003	0.7	B*15:03g	0.003	0.7	C*06:02	0.086	16.4	DRB1*04:04	0.047	9.4	DQB1*05:02:01	0.005	1.0
A*11:01g	0.070	13.1	B*15:07	0.002	0.3	C*07:01g	0.190	35.2	DRB1*04:05:01	0.002	0.3	DQB1*05:03:01	0.017	3.4
A*23:01g	0.018	3.7	B*15:16	0.002	0.3	C*07:02g	0.156	27.9	DRB1*04:06	0.002	0.3	DQB1*05:04	0.002	0.3
A*24:02g	0.069	13.8	B*15:17	0.003	0.7	C*07:04g	0.008	1.7	DRB1*04:07:01	0.012	2.3	DQB1*06:01	0.010	2.0
A*24:03g	0.002	0.3	B*15:18	0.003	0.7	C*08:02	0.032	6.4	DRB1*04:08	0.005	1.0	DQB1*06:02:01	0.149	27.5
A*25:01g	0.022	4.4	B*18:01g	0.045	9.1	C*08:03:01	0.002	0.3	DRB1*07:**01**:**01**	0.156	28.9	DQB1*06:03:01	0.055	11.1
A*26:01g	0.020	4.0	B*27:05g	0.042	8.1	C*12:02	0.010	2.0	DRB1*08:01	0.013	2.7	DQB1*06:04g	0.022	4.4
A*29:01g	0.003	0.7	B*35:01g	0.032	6.4	C*12:03	0.047	9.4	DRB1*08:03:02	0.003	0.7	DQB1*06:09	0.017	3.0
A*29:02:01	0.032	6.4	B*35:02:01	0.003	0.7	C*14:02:01	0.010	2.0	DRB1*09:01:02	0.012	2.3		1.000	
A*30:01g	0.007	1.3	B*35:03g	0.012	2.3	C*15:02g	0.023	4.7	DRB1*10:01:01	0.007	1.3			
A*30:02	0.010	2.0	B*35:08:01	0.003	0.7	C*15:05	0.003	0.7	DRB1*11:01	0.034	6.4			
A*31:01g	0.029	5.7	B*37:01:01	0.018	3.7	C*16:01:01	0.045	9.1	DRB1*11:02:01	0.002	0.3			
A*32:01	0.027	5.4	B*38:01:01	0.010	2.0	C*16:02	0.003	0.7	DRB1*11:03	0.008	1.7			
A*33:01	0.003	0.7	B*39:01g	0.007	1.3	C*17:01g	0.002	0.3	DRB1*11:04:01	0.008	1.7			
A*33:**03g**	0.003	0.7	B*39:06:02	0.002	0.3		1.000		DRB1*12:01g	0.010	2.0			
A*66:01g	0.005	1.0	B*40:01g	0.060	11.4				DRB1*13:01:01	0.045	9.1			
A*68:01g	0.018	3.7	B*40:02g	0.005	1.0				DRB1*13:02:01	0.039	7.0			
A*68:02	0.007	1.3	B*41:01	0.002	0.3				DRB1*13:03:01	0.003	0.7			
A*74:03	0.002	0.3	B*41:02:01	0.002	0.3				DRB1*13:05:01	0.003	0.7			
			B*44:02g	0.101	19.1				DRB1*14:01g	0.017	3.4			
			B*44:03	0.055	11.1				DRB1*15:**01**	0.159	28.9			
			B*44:04	0.002	0.3				DRB1*15:02:01	0.010	2.0			
			B*44:05	0.002	0.3				DRB1*16:01:01	0.002	0.3			
			B*45:01g	0.005	1.0				DRB1*16:02:01	0.003	0.7			
			B*47:01	0.005	1.0					1.000				
			B*48:01g	0.002	0.3									
			B*49:01	0.007	1.3									
			B*50:01	0.017	3.4									
			B*50:02	0.002	0.3									
			B*51:01g	0.047	9.4									
			B*52:01g	0.010	2.0									
			B*53:01:01	0.005	1.0									
			B*55:01	0.023	4.7									
			B*56:01g	0.008	1.7									
			B*57:**01**:**01**	0.037	7.0									

AF, allele frequency; GF, genotype frequency (percentage of individuals carrying the allele). Alleles associated with DILI are shown in bold. Generic codes for ambiguities are denoted with the suffix 'g' (Table S5 in Additional file 1).

### Hardy-Weinberg equilibrium

We used the exact test to determine deviation from Hardy-Weinberg proportions in the ENW population based on the Arlequin's implementation included in the PyPop software (Table 2) [37].

### Haplotype frequencies and linkage disequilibrium

Based on the high LD, which is present at the HLA loci in our cohort (Table S3 in Additional file 1), we selected four haplotypic combinations: HLA-A:HLA-B, HLA-B:HLA-C, HLA-B:HLA-DRB1, HLA-DRB1:HLA-DQB1. Additionally, we included the HLA-DQB1:HLA-DQA1 haplotype, which has been shown to be in LD previously [38].

**Figure 2:**
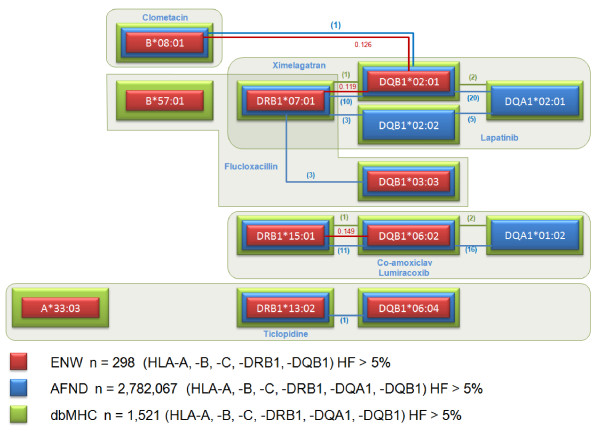
**Linkage disequilibrium and haplotype structure for HLA alleles associated with drug-induced liver injury in Caucasians**. From the literature, we selected class I and class II HLA alleles that have been reported to be associated with DILI. Seven candidate gene and five genome-wide association studies were included in the analysis. Drugs and associated alleles that have been implicated in DILI are organized in frames. Colored boxes represent information gained from the England North West (ENW) cohort (red), dbMHC (green) and AFND (blue). Haplotype frequencies > 5% are depicted by connecting lines. Please note that the extended HLA class II haplotype associated with flucloxacillin-induced DILI comprises B*57:01, DRB1*07:01 and DQB1*03:03, rather than DQB1*02:01. Ambiguous codes A*33:03g, B*08:01g, DQB1*02:01g and DQB1*06:04g in the ENW population were allocated as A*33:03, B*08:01, DQB1*02:01 and DQB1*06:04, respectively. Haplotype frequencies in the ENW population are shown to three decimal places. Values in parentheses indicate the number of populations in dbMHC (green) and AFND (blue) that contain two loci haplotypes with frequencies over 5%. Table S6 in Additional file 1 lists the percentage of individuals from the ENW cohort who are carriers of alleles and haplotypes associated with DILI, as are represented in this figure.

Haplotypes were estimated using maximum likelihood based on Expectation Maximization algorithm [39] at two, three or all loci in the ENW population. We examined haplotype relationships between all alleles associated with DILI. We extended the analysis to include datasets that contain genotype data submitted to dbMHC and also AFND, which is based on frequencies (Figure 2). The pictures were automatically generated using GraphViz software [40].

### Epitope prediction for HLA alleles associated with drug-induced liver injury

In the literature, the following HLA alleles were identified as those that had an association with drug-induced liver toxicity [6-8,11,13,18,20-22]: B*57:01, DRB1*07:01, DQB1*02:01, DRB1*15:01, DQA1*01:02, DQA1*02:01, A*33:03, DRB1*13 and B*08. We wished to test the hypothesis that there is an underlying structural or functional feature shared between these alleles that caused liver toxicity to result under different drug treatments.

A variety of software packages are capable of epitope prediction for HLA alleles. These packages are able to predict the binding affinity for a given HLA allele and a given peptide sequence. In independent trials, one of the leading software packages for epitope prediction is NetMHCpan/NetMHCIIpan [41,42] (for class I and II HLA alleles), which is available as a web-server and an open-source download. We installed a local copy of NetMHCpan and used it to predict the binding affinity for a range of peptides to see if common patterns emerged amongst the liver toxicity-associated alleles, except for the DQA1 alleles; insufficient data were available to make reliable predictions for these alleles, since they require a partner DQB1 allele.

We selected a set of 'control' alleles at the different loci by determining the ten most common HLA-A, -B, -C and -DRB1 alleles in Caucasian populations [33], excluding the 'test alleles' under the assumption that the liver toxicity reaction is rare and hence the mechanism is not present in the most common genetic background.

In the first analysis, we selected 10,000 random human peptides of length 9 amino acids (from the human International Protein Index database v3.80) and ran the NetMHCpan predictors for the class I DILI-associated and control alleles. We also generated 10,000 random peptides of length 15 amino acids (since this is considered optimal for class II alleles) and ran the NetMHCIIpan predictors for the class II DILI-associated and control alleles.

For each combination of peptide sequence and allele, the software produces an affinity score from 0 to 1, where 0 indicates very low affinity and 1 indicates very strong affinity. A score of around 0.4 indicates the peptide is likely to be a weak binder for the allele, and 0.6 a strong binder. The predictions are based on motifs, defined by knowledge of experimental peptide sequences shown to bind each HLA molecule.

For each allele, we obtained 10,000 data points on peptide binding affinity. Following methods defined in the NetMHCpan publications [41,42], we calculated the similarity between all pairs of alleles using a Pearson correlation, for each pairwise comparison of 10,000 data points. The correlation coefficients were converted into a distance matrix, by taking 1-correlation - that is, a correlation of 1 would give a distance of zero. The distance matrix was applied to the neighbor joining algorithm in PHYLIP (version 3.69) [43] to produce distance trees. The trees were then visualized using Molecular Evolutionary Genetics Analysis (version 4.0) [44] and annotations of the DILI-associated alleles were added manually.

## Results

### Study subjects for the HLA-typed archive

Healthy unrelated volunteers (*n *= 400) were recruited from North West England between August 2009 and April 2010. Both DNA and lymphocytes were isolated from 385 individuals. The average number of lymphocytes isolated from each volunteer was 96 million ± 40 million. Gender representation among 400 volunteers was 64% female and 36% male. The average age of volunteers was 29 years (± 10 years, range 18 to 60). The high proportion of volunteers below the age of 30 was due to a high number of university students recruited.

Volunteers from varied ethnic backgrounds were particularly sought, in order to increase the HLA allelic diversity and include low-frequency alleles within the cohort. The ethnicity of individuals was self-reported and included information on ancestry of parents and grandparents. Most volunteers were Caucasians (77.4%; Table S1 in Additional file 1).

Many different HLA alleles were detected as expected in our cohort: 43 different HLA-A alleles, the most frequent of these being HLA-A*02:01, which was present in 176 individuals (23 homozygotes, 153 heterozygotes). The HLA-B locus was the most polymorphic, with 75 different alleles detected, of which the most common allele was B*07:02 (9 homozygotes and 85 heterozygotes). In HLA-C, a total of 29 alleles were present, with the most prevalent being HLA-C*07:01 (9 homozygotes, 110 heterozygotes). The HLA-DRB1 locus contained 42 alleles, with the most common allele being DRB1*15:01, which was present in 110 individuals (10 homozygous, 100 heterozygous). Nineteen different HLA-DQB1 alleles were present, the most frequent of which was DQB1*02:01, carried by 163 individuals (21 homozygotes, 142 heterozygotes). The total number of individuals who were carriers of HLA alleles associated with a range of ADRs is summarized in Table 1. Allele frequencies in Caucasians recruited to donate blood to the HLA-typed archive (*n *= 298) are shown in Table 3. HLA allele genotypes and frequencies were submitted to the AFND and are available for free consultation (population named 'England North West (ENW)' [34].

### HLA allele frequencies and linkage disequilibrium in Caucasians

HLA allele frequency analysis was performed using our HLA-typed cohort and two public repositories, AFND [34] and dbMHC [35].

Most studies that have reported an association between HLA alleles and DILI have been conducted in Caucasians. Therefore, our initial analysis was performed in Caucasians only. To verify the quality of genotyping, the Hardy-Weinberg test was applied to all HLA loci (Table S2 in Additional file 1). All HLA loci were in Hardy-Weinberg equilibrium (*P *> 0.05), which indicates a suitable representation of the individuals sampled [37].

As expected, high LD was observed in several loci of the HLA cohort (Table S3 in Additional file 1), which is concordant with the published literature. The highest LD was detected between haplotypic combinations at the following loci: HLA-DRB1:HLA-DQB1 (D' = 0.95), HLA-B:HLA-C (D' = 0.94), HLA-B:HLA-DRB1 (D' = 0.68), HLA-A:HLA-B (D' = 0.60) and C:DRB1 (D' = 0.60). The HLA-DQB1:HLA-DQA1 haplotype, which had previously been shown to be in LD [38], was also included in the analysis using the AFND and dbMHC datasets.

We compared the frequencies of HLA alleles associated with DILI in Caucasians from our cohort (*n *= 298) with the frequencies in other ethnic groups from AFND and dbMHC. We demonstrated that similar HLA allele frequencies in the Caucasian population exist in all three datasets. Results are summarized according to ethnic background (Table S4a in Additional file 1) and geographic regions (Table S4b, c in Additional file 1). The frequency distribution of HLA alleles associated with DILI across worldwide populations is shown in Figure S1 in Additional file 1.

### HLA alleles associated with DILI and their haplotype structures

We performed an analysis using individual HLA genotype data available from our Caucasian cohort (ENW, *n *= 298). Two loci haplotypes were first estimated in our cohort (ENW) and then the analysis was extended to include datasets on worldwide populations (Figure 2). In order to investigate the LD and haplotype structure of HLA alleles associated with DILI, we performed an analysis using individual HLA genotype data available from our cohort (ENW, *n *= 298) and from the dbMHC database (*n *= 1,521) and haplotype frequency data available for 2,782,067 individuals in the AFND. Haplotypes containing HLA alleles associated with DILI were generated for loci with the highest LD (Table S3 in Additional file 1). We confirmed that LD and the haplotype structure for alleles associated with DILI in our Caucasian cohort correlate well with the data from public repositories.

Two main haplotypes were found and are shown in Figure 2. Two distinct haplotypes in Caucasians contain the following alleles. Haplotype 1 contains alleles associated with a DILI response to lapatinib and ximelagatran, namely DRB1*07:01, DQB1*02:01g and DQA1*02:01. B*08:01g (B8 was reported to be associated with clometacin) is also in strong LD with DQB1*02:01g and in weaker LD with DRB1*07:01. B*57:01, which has been associated with flucloxacillin DILI is in strong LD with DRB1*0701, but interestingly, the extended HLA class II haplotype contains DQB1*03:03 rather than DQB1*02 [6]. Haplotype 2 contains alleles associated with a DILI response to lumiracoxib and co-amoxiclav, such as DRB1*15:01, DQB1*06:02 and DQA1*01:02. Ticlopidine-induced DILI has been linked with the A*33:03g, DRB1*13:02 and DQB1*06:04g alleles, which are common in Japanese populations. However, interestingly, the DQB1*06:04 allele is in strong LD with DQA1*01:02 not only in Asian populations but also in all major worldwide populations, including Caucasians (data not shown). Unfortunately, DQA1*01:02 was not determined in the ticlopidine study.

**Figure 3:**
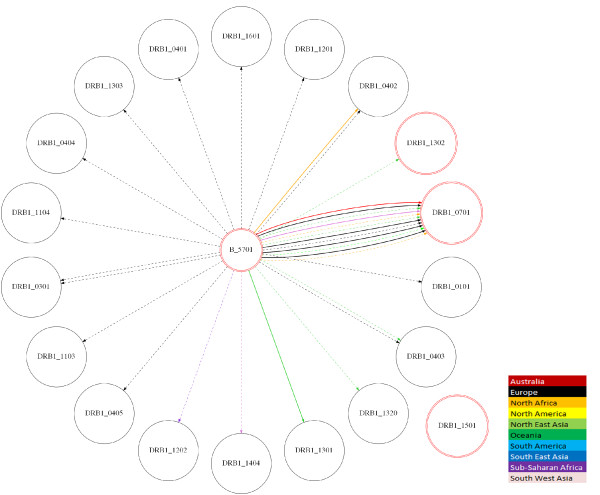
Haplotype combinations between HLA-B*57:01 and HLA-DRB1 alleles. All haplotype combinations (colored lines) of the HLA-B*57:01 allele with HLA-DRB1 alleles reported in the dbMHC database are shown (*n *= 8,569). Haplotype frequencies were estimated using the Expectation Maximization algorithm described in PyPop [37]. Frequencies > 1% are shown in bold whereas frequencies < 1% are represented by dotted lines. Circles in red correspond to alleles that have been associated with DILI. As shown, high LD can be observed between the B*57:01 and DRB1*07:01 alleles, which have been associated with DILI. This picture was automatically generated using the GraphViz software [40] and is available as Scalable Vector Graphics (SVG) file.

It is important to note that, with regards to nomenclature, the 'g' in A*33:03g, B*08:01g, DQB1*02:01g and DQB1*06:04g corresponds to generic codes in which the alleles were not distinguished by sequencing. It is very likely, however, that those alleles correspond to A*33:03, B*08:01, DQB1*02:01 and DQB1*0604 given their higher frequencies compared with other ambiguous alleles. To examine the associations among other ethnicities, we generated images in Scalable Vector Graphics (SVG) using GraphViz [40,45] to demonstrate the relationship among different populations (Figure 3). As expected, we demonstrated strong LD within two groups of HLA alleles previously associated with DILI, indicating the existence of two defined haplotypes. These haplotypes are relevant in several populations.

### Peptide predictions in relation to HLA alleles associated with DILI

**Figure 4:**
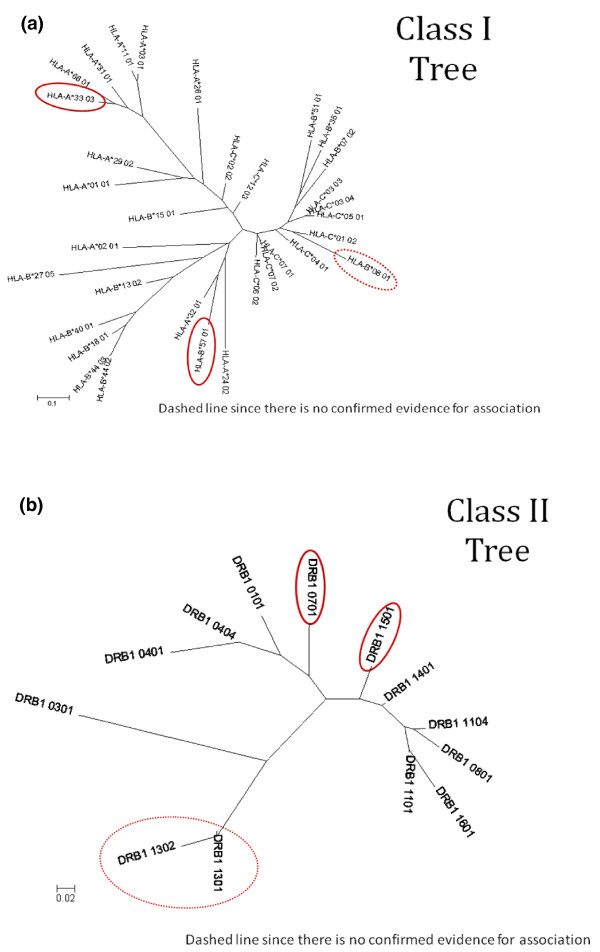
**Distance trees for HLA class I and class II alleles associated with DILI compared with most frequent HLA alleles selected as controls**. **(a) **Class I alleles. **(b) **Class II alleles. Controls were selected based on high frequency alleles in Caucasians. HLA alleles associated with DILI are circled in red.

As detailed in Materials and methods, we generated distance trees, based on predicted binding scores for 10,000 random peptides using the NetMHCpan [41] and NetMHCIIpan [42] software, for the DILI-associated alleles and the most common HLA class I and class II alleles in Caucasian populations. The trees enable us to test if there is any apparent similarity in peptide binding between different alleles that have previously been associated with DILI, which might indicate a common functional pathway. The results indicate that there is no obvious relationship between predicted peptide binding motifs for class I DILI-associated alleles (A*33:03, B*08 and B*57:01) as they are separated by considerable distance on the tree (Figure 4). For class II DILI-associated alleles, DRB1*15:01 and DRB1*07:01, the picture is not as clear, since DRB1*15:01 and DRB1*07:01 are not separated by a large distance in their peptide binding capacity, and thus it is likely that they will present some of the same epitopes to the immune system. It should be noted, however, that DRB1*15:01 is more closely associated with one of the 'control' alleles (DRB1*14:01, for which no DILI association has ever been detected) than with DRB1*07:01.

## Discussion

For many HLA-associated ADRs the causative allele is yet to be identified. Given the strong LD in the MHC region, it is difficult to conclude that HLA alleles that show the strongest association with drug-induced adverse reactions are indeed the causative alleles. These associations encompass a diverse range of drugs and clinical manifestations, such as DILI or delayed type hypersensitivity reactions.

The complex interplay between HLA genes and haplotypes can only be replicated in systems where these haplotypes are present. Humanized animal models, in which specific human HLA alleles can be inserted into the mouse genome, have previously been used to model autoimmunity [46]. However, given the great complexity in the MHC region, where LD can confound the discovery of genetic associations and where there is a likely role for specific HLA haplotypes, it is unclear how successful this approach would be in studying ADRs to low molecular weight drugs in particular. Through the use of human lymphocytes in which HLA haplotypes can be determined, we can paint a more complete picture of the mechanisms underlying drug antigenicity. It is possible that other, as yet undetermined HLA loci or non-HLA genes may be implicated in ADRs that reside on an extended haplotype.

Our primary aim was to establish a HLA-typed DNA archive that could be used together with the existing publicly accessible data repositories for *in silico *HLA analysis. In addition, we established a cell archive from healthy individuals to test the propensity of drugs to elicit an immune response *in vitro*. We therefore recruited 400 individuals from different ethnicities, which was important as some HLA-associations that have been discovered are confined to specific ethnic groups. In order to be able to study the involvement of low-frequency alleles, it was therefore important to recruit individuals from these backgrounds. Given that the strongest associations so far were found between immune-mediated ADRs and HLA alleles, we undertook high-resolution sequence-based typing for five classical HLA loci (HLA-A, -B, -C, -DRB1 and -DQB1) and used the National Marrow Donor Program HLA allele nomenclature to represent ambiguities. The National Marrow Donor Program coding algorithm is used in registries and cord blood banks around the world that collect and store HLA typing information for volunteer donors for bone marrow transplantation and is used to select and prioritize a list of potentially suitable donors within large donor data files [47].

The HLA-typed cell archive will be used to assess the role of HLA haplotypes in drug-induced hypersensitivity. Our cohort sample size calculations were based on ximelagatran, a drug withdrawn from the market because of liver toxicity during phase III studies [11]. Preclinical studies in a variety of species failed to detect liver toxicity associated with ximelagatran. An immune mechanism has been postulated based on the prolonged time to onset of alanine aminotransferase elevation (one to six months), evidence of drug-specific T cells (2 out of 7 occupationally exposed workers with skin symptoms and 2 out of 21 orally treated patients with elevated alanine aminotransferase), the pharmacogenomic MHC association and evidence of ximelagatran binding to soluble HLA-DRB1*07:01 molecules [11].

In order to explore the associations between DILI and HLA class I and class II alleles, we utilized *in silico *approaches to investigate allele and haplotype associations by using individual level data from our archival collection and from public repositories. The latter provided us with a dataset that contained HLA allele frequency information on approximately four million individuals from more than 800 worldwide populations. Firstly, we demonstrated that different HLA alleles found to be associated with DILI are in strong LD. Secondly, in an independent unbiased analysis that included all available HLA alleles, we showed that LD is also strong in populations of non-Caucasian ancestry. Thirdly, we showed that alleles that have been reported to be associated with DILI caused by several chemically unrelated drugs, such as flucloxacillin, ximelagatran, lapatinib and antituberculosis drugs, reside on similar haplotypes. Although some studies reported that drug-induced hepatotoxicity was associated with HLA haplotypes rather than the single HLA alleles [6,7,11,48], due to relatively small number of participants in each study, it has not been possible to analyze extended haplotypes in detail. We report here that DILI caused by at least nine different drugs can be related to two main haplotypes, which are illustrated in Figure 2. Interestingly, for ticlopidine-induced DILI in Japanese patients the strongest association has been detected with A*33:03, which has higher frequency in Asian populations than in Caucasians (Figure S1 in Additional file 1). However, the ancestral haplotype 44.4 in Caucasians, which contains A*33:03, DRB1*13:02 and DQB1*06:04, also contains DQA1*01:02, which has been associated with lumiracoxib- and co-amoxiclav-associated toxicity [7,13,21], in a haplotype containing DQB1*06:02. Our preliminary analysis suggests that the peptide binding capability of DQA1*01:02-DQB1*06:02 and DQA1*01:02-DQB1*06:04 would be the same, as the antigen recognition site of DQB1*06:02 and DQB1*06:04 is identical, and hence it may be that this is a common factor between the DILI associations with ticlopidine and lumiracoxib/co-amoxiclav. The identification of common haplotypes associated with DILI raises the intriguing possibility that common causal alleles within the MHC may predispose to DILI - this may now be amenable to investigation by next-generation sequencing technologies.

Several alleles that have not been implicated in DILI previously have been identified to be in strong LD with liver toxicity-associated alleles. These are A*01:01, B*40:01 and DQA1*01:01. Although they are relatively common, they may give us a valuable clue to the haplotype on which a potential low-frequency or rare (less than three reports worldwide) causal variant resides. It is important to note that another role for our high resolution typed archive is to confirm the existence of reported rare HLA alleles.

It is important to note that the identified haplotypes represent a significant proportion of a general population. It is likely, therefore, that other factors are required in addition to specified HLA allele carriage to elicit the adverse reaction, implying that specified HLA carriage is necessary but not sufficient to elicit this effect. Our HLA-characterized cell archive can be utilized to empirically investigate such alternative hypotheses.

One of the limitations of our analyses is that although DQ alleles are implicated as part of the causal locus in the DILI causing haplotypes for four unrelated drugs, no heterodimer data for DQA1-DQB1 structure-binding relationships are available, which may limit the peptide sequence inferences drawn.

We carried out peptide binding predictions that confirmed that alleles associated with DILI are unrelated. The results clearly indicated that there is no overall similarity in peptide binding across all class I and class II alleles implicated in DILI, compared with the control set of HLA alleles. Within the HLA class I alleles associated with liver toxicity, the alleles were less similar than the control set, indicating that it is unlikely there is a shared mechanism of peptide binding. Within the class II set, the two DILI-associated alleles (DRB1*07:01, DRB1*15:01) were, however, slightly more similar to each other than they were to the control set.

## Conclusions

The present study demonstrates how creating a DNA biobank generated from healthy volunteers can be used to study HLA-associated ADRs. The HLA-typed cells stored in our cell bank will be an invaluable tool in functional assays to determine the propensity of different drugs to elicit a T-cell response in future studies. Our knowledge of HLA LD and haplotype structure in Caucasians and in other worldwide populations can help selecting cells of certain genotypes to be used as positive and negative controls in immunological studies.

## Abbreviations

AFND: Allele Frequency Net Database; ADR: adverse drug reaction; dbMHC: Major Histocompatibility Complex Database; DILI: drug-induced liver injury; ENW: England North West; HLA: human leukocyte antigen; LD: linkage disequilibrium; MHC: major histocompatibility complex; p-i concept: pharmacological interaction with immune receptors.

## Competing interests

The authors declare that they have no competing interests.

## Authors' contributions

AA made substantial contributions to conception, design, analysis and interpretation of data, drafted the manuscript and gave final approval of the version to be published. FGG participated in the design of the study, performed the analyses and revised the manuscript critically for important intellectual content. ARJ carried out the protein bioinformatics analyses, and was involved in drafting the manuscript and revising it critically. BKP and MP participated in the design of the study, interpretation of data and critically revised the manuscript. CB, KM, VP and ML carried out the HLA-typed cell archive data analyses and drafted the manuscript. JB and JE recruited volunteers and carried out clinical data analyses. KC, DJN and NF participated in study design and coordination. All authors read and approved the final manuscript.

## Supplementary Material

Additional file 1 **Supplementary tables and figures**. Table S1: number of individuals and their ethnicities from three different datasets used in the haplotype analyses: (a) England North West; (b) Allele Frequency Net Database (AFND); (c) dbMHC database. Table S2: Hardy-Weinberg proportions calculated for five loci: HLA-A, -B, -C, -DRB1 and -DQB1. Table S3: LD values estimated by the PyPop software using different statistics. Table S4: distribution of HLA alleles associated with drug-induced liver injury by: (a) major ethnic groups in the AFND; (b) geographic region in the AFND; and (c) geographic region in dbMHC. Table S5: list of alleles for each allele with suffix 'g'. Table S6: counts (frequencies) of Caucasian individuals from the ENW archive (*n *= 298) who carry alleles and two loci haplotypes implicated in DILI. Figure S1: alleles associated with DILI selected from the AFND and their frequencies. Click here for file
