# Supplementary material for: In silico analysis of HLA associations with drug-induced liver injury: use of a HLA-genotyped DNA archive from healthy volunteers
Source: Genome Med. 2012 Jun 25;4(6):51. doi: 10.1186/gm350 (PMC3698530; doi:10.1186/gm350)
Supplement: Additional file 1 — Supplementary tables and figures. Table S1: number of individuals and their ethnicities from three different datasets used in the haplotype analyses: (a) England North West; (b) Allele Frequency Net Database (AFND); (c) dbMHC database. Table S2: Hardy-Weinberg proportions calculated for five loci: HLA-A, -B, -C, -DRB1 and -DQB1. Table S3: LD values estimated by the PyPop software using different statistics. Table S4: distribution of HLA alleles associated with drug-induced liver injury by: (a) major ethnic groups in the AFND; (b) geographic region in the AFND; and (c) geographic region in dbMHC. Table S5: list of alleles for each allele with suffix 'g'. Table S6: counts (frequencies) of Caucasian individuals from the ENW archive (n = 298) who carry alleles and two loci haplotypes implicated in DILI. Figure S1: alleles associated with DILI selected from the AFND and their frequencies. [file gm350-S1.DOC]

Additional file

**Table s1 Datasets used for the analysis**

1. **England North West (n=385)**

| Ethnic origin | Individuals | Percentage (%) |
| --- | --- | --- |
| Caucasian | 298 | 77.4 |
| Indian | 28 | 7.3 |
| Other | 39 | 10.1 |
| Chinese | 15 | 3.9 |
| Black | 5 | 1.3 |
| Total | 385 (100) | 100 |

**“Other” ethnicity mainly shows mixed ethnicity (European and Asian, African or Chinese). As parents and grandparents ethnicity was included in the questionnaire, in some cases it was difficult to ascertain self-declared ethnicity.**

1. **Allele Frequency Net Database by ethnicity**

| Ethnic origin | Populations | Individuals |
| --- | --- | --- |
| Amerindian | 84 | 6138 |
| Arab | 29 | 3691 |
| Asian | 54 | 7096 |
| Australian Aboriginal | 5 | 587 |
| Austronesian | 20 | 2811 |
| Black | 79 | 426496 |
| Caucasoid | 237 | 2782067 |
| Melanesian | 29 | 1892 |
| Mestizo | 21 | 2324 |
| Micronesian | 2 | 129 |
| Oriental | 179 | 124000 |
| Persian | 8 | 6918 |
| Polynesian | 17 | 1054 |
| Siberian | 22 | 1274 |
| Other | 17 | 24913 |
| Total | 803 | 3391390 |

1. **dbMHC database (n=8569)**

| Region | Name | Sample | A | B | C | DRB1 | DQA1 | DQB1 | DPA1 | DPB1 |
| --- | --- | --- | --- | --- | --- | --- | --- | --- | --- | --- |
| AUS | Cape_York | 83 | 83 | 83 | 83 | 83 | 83 | 83 |  | 83 |
| AUS | Groote_Eylandt | 73 | 73 | 73 | 73 |  |  |  |  |  |
| AUS | Kimberley | 24 | 24 | 24 | 24 | 24 | 24 | 24 |  | 24 |
| AUS | Yuendumu | 190 | 190 | 190 | 190 |  |  |  |  |  |
| EUR | Croatian | 149 | 149 | 149 |  |  |  |  |  |  |
| EUR | Czech | 96 | 96 | 96 | 96 | 96 | 96 | 96 |  | 96 |
| EUR | Finn | 90 | 90 | 90 | 90 | 90 |  |  |  |  |
| EUR | Georgian | 103 | 103 | 103 | 103 |  |  |  |  |  |
| EUR | Irish | 983 | 983 | 983 | 983 | 983 |  |  |  |  |
| EUR | Slovenian | 100 |  |  |  | 100 | 100 | 100 | 100 | 100 |
| NAF | Algerian | 99 |  |  |  | 99 |  | 99 |  |  |
| NAF | Chaouya | 56 | 56 | 56 |  | 56 |  |  |  |  |
| NAF | Metalsa | 56 | 56 | 56 |  | 56 |  |  |  |  |
| NAF | Moroccan1 | 98 |  |  |  | 98 | 98 | 98 |  |  |
| NAF | Moroccan2 | 85 |  |  |  | 85 | 85 | 85 |  |  |
| NAM | Amerindian | 229 | 229 | 229 | 229 |  |  |  |  |  |
| NAM | Canoncito | 40 | 40 | 40 | 40 | 40 | 40 | 40 |  | 40 |
| NAM | Lacandon | 162 |  |  |  | 162 | 162 | 162 |  |  |
| NAM | Pima | 79 | 79 | 79 |  |  |  |  | 79 | 79 |
| NAM | Seri | 25 | 25 | 25 |  | 25 | 25 | 25 | 25 |  |
| NAM | Sioux | 80 |  |  |  | 80 | 80 | 80 |  | 80 |
| NAM | Yupik | 251 | 251 | 251 |  | 251 |  | 251 |  |  |
| NAM | Zuni | 50 |  |  |  | 50 | 50 | 50 |  | 50 |
| NEA | Buriat | 140 | 140 |  |  |  |  |  |  |  |
| NEA | Korean | 199 | 199 | 199 | 199 | 199 |  |  |  |  |
| NEA | Okinawan | 104 | 104 | 104 | 104 |  |  |  |  |  |
| NEA | Ryukuan | 142 | 142 |  |  |  |  |  |  |  |
| NEA | Tuva | 164 | 164 | 164 | 164 | 164 |  |  |  |  |
| OCE | American_Samoa | 50 | 50 | 50 | 50 |  |  |  |  |  |
| OCE | East_Timorese | 49 | 49 |  |  | 49 | 49 | 49 | 49 | 49 |
| OCE | Filipino | 94 | 94 | 94 | 94 | 94 |  | 94 | 94 | 94 |
| OCE | Indonesian | 49 | 49 | 49 | 49 |  |  |  |  |  |
| OCE | Ivatan | 25 | 25 | 25 | 25 | 25 |  |  |  |  |
| OCE | Moluccan | 23 | 23 |  |  | 23 | 23 | 23 | 23 | 23 |
| OCE | PNG_Highlander | 60 | 60 | 60 |  | 60 | 60 | 60 | 60 | 60 |
| OCE | PNG_Lowlander1 | 48 |  |  |  |  | 48 | 48 | 48 | 48 |
| OCE | PNG_Lowlander2 | 66 | 66 |  |  | 66 |  | 66 |  |  |
| SAM | Bari | 16 | 16 | 16 | 16 |  |  |  |  |  |
| SAM | Kaiowa | 136 | 136 | 136 | 136 | 136 | 136 | 136 |  |  |
| SAM | Nandewa | 50 | 50 | 50 | 50 | 50 | 50 | 50 |  |  |
| SAM | Panama | 50 |  |  |  | 50 | 50 | 50 |  | 50 |
| SAM | Ticuna | 46 |  |  |  | 46 | 46 | 46 | 46 | 46 |
| SEA | Ami | 63 | 63 | 63 | 63 | 63 |  |  |  |  |
| SEA | Atayal | 106 | 106 | 106 | 106 | 106 |  |  |  |  |
| SEA | Bunun | 101 | 101 | 101 | 101 | 101 |  |  |  |  |
| SEA | Hakka | 50 | 50 | 50 | 50 | 50 |  |  |  |  |
| SEA | Han1 | 149 | 149 | 149 |  |  |  |  |  |  |
| SEA | Han2 | 569 | 569 | 569 |  |  |  |  |  |  |
| SEA | Kinh | 99 |  |  |  | 99 |  | 99 |  |  |
| SEA | Malay | 92 | 92 | 92 | 92 |  |  |  |  |  |
| SEA | Muong | 63 |  |  |  | 63 |  | 63 |  |  |
| SEA | Paiwan | 50 | 50 | 50 | 50 | 50 |  |  |  |  |
| SEA | Puyuma | 31 | 31 | 31 | 31 | 31 |  |  |  |  |
| SEA | Rukai | 50 | 50 | 50 | 50 | 50 |  |  |  |  |
| SEA | Saisiat | 51 | 51 | 51 | 51 | 51 |  |  |  |  |
| SEA | Thai | 92 | 92 | 92 | 92 |  |  |  |  |  |
| SEA | Toroko | 55 | 110 | 110 | 110 | 110 |  |  |  |  |
| SEA | Tsou | 51 | 51 | 51 | 51 | 51 |  |  |  |  |
| SEA | Yami | 39 | 39 | 39 | 39 | 39 |  |  |  |  |
| SSA | Doggon | 87 | 87 | 87 | 87 | 87 |  |  |  |  |
| SSA | Kenyan | 84 | 84 | 84 | 84 |  | 84 | 84 | 84 | 84 |
| SSA | Kenyan_Highlander | 240 | 240 | 240 | 240 |  |  |  |  |  |
| SSA | Kenyan_Lowlander | 265 | 265 | 265 | 265 |  |  |  |  |  |
| SSA | Mandenka | 93 | 93 | 93 |  |  |  |  |  |  |
| SSA | Rwandan | 197 |  |  |  | 197 |  | 197 |  |  |
| SSA | Shona | 152 | 152 | 152 | 152 | 152 | 152 | 152 |  | 152 |
| SSA | Ugandan | 159 | 159 | 159 | 159 |  |  |  |  |  |
| SSA | Zambian | 43 | 43 | 43 | 43 |  |  |  |  |  |
| SSA | Zulu1 | 82 | 82 | 82 | 82 |  |  |  |  |  |
| SSA | Zulu2 | 85 |  |  |  | 85 | 85 | 85 |  | 85 |
| SWA | Druze | 99 | 99 | 99 | 99 |  |  |  |  |  |
| SWA | Kurdish | 28 | 28 | 28 | 28 |  |  |  |  |  |
| SWA | New_Delhi | 53 | 53 | 53 | 53 |  |  |  |  |  |
| SWA | Omani | 118 | 118 | 118 |  |  |  |  |  |  |
| SWA | South_Indian | 72 | 72 | 72 |  | 72 |  |  |  |  |
| SWA | Tamil | 47 | 47 | 47 | 47 |  |  |  |  |  |
| SWA | Turk | 242 |  |  |  | 242 | 242 | 242 |  |  |
| Pops: 77 |  | 8569 | 7120 | 6700 | 5023 | 5039 | 1868 | 2737 | 608 | 1243 |

AUS=Australia, EUR=Europe, NAF=North Africa, NAM, North America, NEA=North East Asia, OCE=Oceania, SAM=South and Central America, SEA=South East Asia, SSA=Sub-Saharan Africa, SWA=South West Africa.

**Table s2. Test of deviation of Hardy-Weinberg Proportions (HWP)**

| Locus | Type | Observed | Expected | Χ^2^ | p-value |
| --- | --- | --- | --- | --- | --- |
| A | Hmz | 39 | 47.59 | 1.55 | 0.2132 |
|  | Htz | 259 | 250.41 | 0.29 | 0.5874 |
| B | Hmz | 19 | 23.4 | 0.83 | 0.3634 |
|  | Htz | 279 | 274.6 | 0.07 | 0.7908 |
| C | Hmz | 28 | 30.01 | 0.14 | 0.7132 |
|  | Htz | 270 | 267.99 | 0.02 | 0.9021 |
| DRB1 | Hmz | 33 | 30.67 | 0.18 | 0.6739 |
|  | Htz | 265 | 267.33 | 0.02 | 0.8867 |
| DQB1 | Hmz | 47 | 45.83 | 0.03 | 0.8632 |
|  | Htz | 251 | 252.17 | 0.01 | 0.9415 |

Hmz=Homozygosity, Htz=Heterozygosity.

**Table s3. Linkage disequilibrium (LD) in Caucasian individuals from the HLA-typed archive**

| HLA locus pair |  | D’ | Wn | ln(L_1) | ln(L_0) | S |
| --- | --- | --- | --- | --- | --- | --- |
| **A:B** |  | **0.60306** | 0.49596 | -2287.59 | -2734.75 | 894.32 |
| A:C |  | 0.53484 | 0.42325 | -2171.13 | -2491.76 | 641.25 |
| A:DRB1 |  | 0.46282 | 0.36034 | -2294.82 | -2550.41 | 511.19 |
| A:DQB1 | | 0.39678 | 0.28053 | -2103.49 | -2256.89 | 306.79 |
| **B:C** |  | **0.93784** | 0.84463 | -1789.32 | -2887.37 | 2196.11 |
| **B:DRB1** |  | **0.67516** | 0.50906 | -2385.65 | -2946.03 | 1120.75 |
| **B:DQB1** | | **0.63109** | 0.56267 | -2239.70 | -2652.50 | 825.61 |
| **C:DRB1** |  | **0.60037** | 0.46283 | -2286.27 | -2703.04 | 833.53 |
| C:DQB1 | | 0.54114 | 0.45580 | -2123.23 | -2409.51 | 572.57 |
| **DRB1:DQB1** | | **0.95138** | 0.83858 | -1532.92 | -2468.17 | 1870.50 |

Highest LD values are shown in bold**.** LD based on Hendrick’s D’ statistic (D’), LD based on Cramer V statistic (Wn), pair log-likelihoods (ln(L_1) and ln(L_0)) and S statistic were estimated using the PyPop Software .

**Table s4 Frequency distribution of DILI HLA alleles by ethnic group and geographic region**

1. **Distribution of DILI HLA alleles by major ethnic groups in the AFND**

| Allele | ENW  n=298 | AME | ARA | ASI | ABO | AUS | BLA | CAU | MEL | MES | MIC | ORI | PER | POL | SIB |
| --- | --- | --- | --- | --- | --- | --- | --- | --- | --- | --- | --- | --- | --- | --- | --- |
| A*33:03 | 0.003 | 0.005 | 0.012 | 0.097 | 0.002 | 0.085 | 0.041 | 0.005 | - | 0.012 | - | 0.081 | 0.056 | 0.019 | 0.071 |
|  |  | *n=489* | *n=605* | *n=3496* | *n=294* | *n=258* | *n=6166* | *n=15088* |  | *n=41* |  | *n=7188* | *n=100* | *n=106* | *n=169* |
| B*08:01 | 0.149 | 0.011 | 0.058 | 0.018 | 0.014 | 0.000 | 0.039 | 0.101 | - | 0.018 | - | 0.004 | 0.076 | - | 0.008 |
|  |  | n=1772 | n=855 | n=3103 | n=369 | n=50 | n=7383 | n=17320 |  | n=144 |  | n=5346 | n=100 |  | n=169 |
| B*57:01 | 0.037 | 0.006 | 0.011 | 0.022 | 0.006 | 0.020 | 0.008 | 0.033 | - | 0.012 | - | 0.006 | 0.010 | - | 0.033 |
|  |  | *n=1075* | *n=751* | *n=2923* | *n=369* | *n=183* | *n=5502* | *n=16730* |  | *n=41* |  | *n=3942* | *n=100* |  | *n=169* |
| DRB1*07:01 | 0.156 | 0.015 | 0.143 | 0.127 | 0.040 | 0.049 | 0.089 | 0.122 | 0.002 | 0.070 | - | 0.046 | 0.095 | 0.030 | 0.063 |
|  |  | *n=2296* | *n=2044* | *n=4216* | *n=103* | *n=698* | *n=5912* | *n=24983* | *n=707* | *n=473* |  | *n=9578* | *n=338* | *n=490* | *n=1274* |
| DRB1*13:02 | 0.039 | 0.005 | 0.048 | 0.028 | - | 0.013 | 0.075 | 0.042 | 0.000 | 0.027 | 0.008 | 0.043 | 0.028 | 0.012 | 0.033 |
|  |  | *n=2557* | *n=1792* | *n=4638* |  | *n=893* | *n=5710* | *n=24842* | *n=220* | *n=482* | *n=129* | *n=10661* | *n=231* | *n=428* | *n=835* |
| DRB1*15:01 | 0.159 | 0.014 | 0.053 | 0.081 | 0.045 | 0.068 | 0.026 | 0.127 | 0.150 | 0.055 | 0.058 | 0.076 | 0.055 | 0.029 | 0.068 |
|  |  | *n=1848* | *n=1945* | *n=4144* | *n=103* | *n=792* | *n=4641* | *n=23859* | *n=1560* | *n=482* | *n=129* | *n=11225* | *n=338* | *n=289* | *n=1274* |
| DQA1*01:02 | - | 0.016 | 0.125 | 0.113 | 0.110 | 0.130 | 0.324 | 0.178 | 0.301 | 0.080 | 0.232 | 0.153 | - | 0.065 | 0.075 |
|  |  | *n=2444* | *n=453* | *n=1792* | *n=144* | *n=62* | *n=1850* | *n=8035* | *n=332* | *n=355* | *n=129* | *n=3519* |  | *n=238* | *n=1031* |
| DQA1*02:01 | - | 0.017 | 0.186 | 0.213 | 0.040 | 0.110 | 0.083 | 0.127 | 0.004 | 0.070 | 0.000 | 0.048 | 0.106 | 0.021 | 0.060 |
|  |  | *n=1972* | *n=549* | *n=1792* | *n=103* | *n=62* | *n=1850* | *n=9335* | *n=332* | *n=411* | *n=129* | *n=4439* | *n=396* | *n=238* | *n=1221* |
| DQB1*02:01 | 0.273 | 0.029 | 0.250 | 0.156 | 0.071 | 0.081 | 0.198 | 0.204 | 0.006 | 0.134 | 0.000 | 0.077 | 0.227 | 0.024 | 0.089 |
|  |  | *n=2490* | *n=1291* | *n=3893* | *n=103* | *n=471* | *n=4810* | *n=10278* | *n=524* | *n=476* | *n=129* | *n=5701* | *n=396* | *n=417* | *n=570* |
| DQB1*06:02 | 0.149 | 0.013 | 0.060 | 0.039 | 0.040 | 0.031 | 0.203 | 0.102 | 0.118 | 0.058 | 0.050 | 0.051 | 0.085 | 0.034 | 0.075 |
|  |  | *n=3249* | *n=1596* | *n=4112* | *n=103* | *n=471* | *n=5746* | *n=12293* | *n=552* | *n=588* | *n=129* | *n=6610* | *n=396* | *n=667* | *n=1274* |
| DQB1*06:04 | 0.022 | 0.005 | 0.039 | 0.014 | - | 0.003 | 0.036 | 0.027 | 0.000 | 0.013 | 0.008 | 0.032 | 0.037 | 0.010 | 0.013 |
|  |  | *n=1915* | *n=1596* | *n=3952* |  | *n=167* | *n=5482* | *n=10664* | *n=28* | *n=588* | *n=129* | *n=5477* | *n=396* | *n=199* | *n=888* |

AME=Amerindian, ARA=Arab, ASI=Asian, ABO=Aust Aboriginal, AUS=Austronesian, BLA=Black, CAU=Caucasian, MEL=Melanesian, MES=Mestizo, MIC=Micronesian, ORI=Oriental, PER=Persian, POL=Polynesian, SIB=Siberian. (-) No data available.

Frequencies in red show the highest frequency of the allele.

1. **Distribution of DILI HLA alleles by geographic region in the AFND**

| Allele | ENW n=298 | NAFR | WEUR | EEUR | MIDE | SAFR | ASIA | PACI | AUST | NAME | SCAM |
| --- | --- | --- | --- | --- | --- | --- | --- | --- | --- | --- | --- |
| A*33:03 | 0.003 | 0.015 | 0.004 | 0.008 | 0.032 | 0.023 | 0.083 | 0.013 | 0.002 | 0.039 | 0.035 |
|  |  | *n=471* | *n=10856* | *n=1184* | *n=678* | *n=2493* | *n=8744* | *n=156* | *n=428* | *n=12203* | *n=315* |
| B*08:01 | 0.149 | 0.052 | 0.105 | 0.058 | 0.052 | 0.037 | 0.007 | 0.000 | 0.056 | 0.048 | 0.039 |
|  |  | *n=575* | *n=11965* | *n=1194* | *n=824* | *n=2690* | *n=6185* | *n=50* | *n=503* | *n=15575* | *n=506* |
| B*57:01 | 0.037 | 0.016 | 0.034 | 0.018 | 0.009 | 0.012 | 0.011 | - | 0.004 | 0.018 | 0.012 |
|  |  | *n=373* | *n=11392* | *n=1244* | *n=824* | *n=1373* | *n=4699* |  | *n=503* | *n=14316* | *n=401* |
| DRB1*07:01 | 0.156 | 0.155 | 0.121 | 0.089 | 0.128 | 0.080 | 0.070 | 0.019 | 0.040 | 0.103 | 0.068 |
|  |  | *n=1417* | *n=15234* | *n=1631* | *n=2162* | *n=1833* | *n=13545* | *n=1483* | *n=103* | *n=18295* | *n=2630* |
| DRB1*13:02 | 0.039 | 0.053 | 0.040 | 0.035 | 0.049 | 0.097 | 0.038 | 0.008 | - | 0.044 | 0.026 |
|  |  | *n=1106* | *n=15125* | *n=1662* | *n=2074* | *n=1783* | *n=14539* | *n=1283* |  | *n=18260* | *n=2783* |
| DRB1*15:01 | 0.159 | 0.057 | 0.135 | 0.070 | 0.041 | 0.012 | 0.078 | 0.117 | 0.045 | 0.083 | 0.033 |
|  |  | *n=1220* | *n=14093* | *n=1809* | *n=2322* | *n=602* | *n=15139* | *n=2276* | *n=103* | *n=17726* | *n=2720* |
| DQA1*01:02 | - | 0.167 | 0.175 | 0.209 | 0.125 | 0.338 | 0.132 | 0.202 | 0.110 | 0.081 | 0.083 |
|  |  | *n=296* | *n=4127* | *n=1763* | *n=1078* | *n=1403* | *n=7741* | *n=761* | *n=144* | *n=1768* | *n=2456* |
| DQA1*02:01 | - | 0.170 | 0.144 | 0.083 | 0.151 | 0.079 | 0.093 | 0.017 | 0.040 | 0.067 | 0.067 |
|  |  | *n=392* | *n=5207* | *n=2046* | *n=1574* | *n=1403* | *n=8788* | *n=761* | *n=103* | *n=1383* | *n=2627* |
| DQB1*02:01 | 0.273 | 0.246 | 0.245 | 0.122 | 0.219 | 0.161 | 0.110 | 0.018 | 0.071 | 0.152 | 0.082 |
|  |  | *n=1125* | *n=6444* | *n=1934* | *n=1421* | *n=1411* | *n=8623* | *n=1205* | *n=103* | *n=10436* | *n=2445* |
| DQB1*06:02 | 0.149 | 0.082 | 0.106 | 0.076 | 0.049 | 0.218 | 0.055 | 0.065 | 0.040 | 0.102 | 0.055 |
|  |  | *n=910* | *n=7717* | *n=2594* | *n=2239* | *n=2057* | *n=10622* | *n=1483* | *n=103* | *n=11071* | *n=3176* |
| DQB1*06:04 | 0.022 | 0.030 | 0.025 | 0.025 | 0.040 | 0.064 | 0.026 | 0.007 | - | 0.021 | 0.022 |
|  |  | *n=910* | *n=6623* | *n=2543* | *n=2079* | *n=1813* | *n=8357* | *n=418* |  | *n=9484* | *n=2946* |

NAFR=North Africa, WEUR=Western Europe, EEUR=Eastern Europe, MIDE=Middle East, SAFR=Sub-Saharan Africa, ASIA=Asia, PACI=Pacific Islands, AUST=Australia, NAME=North America, SCAM=South and Central America. (-) No data available.

Frequencies in red show the highest frequency of the allele.

1. Distribution of DILI HLA alleles by geographic region in the dbMHC database

| Allele | ENW n=298 | AUS | EUR | NAF | NAM | NEA | OCE | SAM | SEA | SSA | SWA |
| --- | --- | --- | --- | --- | --- | --- | --- | --- | --- | --- | --- |
| A*33:03 | 0.003 | - | 0.004 | 0.018 | 0.004 | 0.088 | 0.080 | - | 0.106 | 0.025 | 0.039 |
|  |  |  | *n=1235* | *n=56* | *n=229* | *n=467* | *n=143* |  | *n=952* | *n=1162* | *n=389* |
| B*08:01 | 0.149 | 0.029 | 0.125 | 0.085 | 0.019 | 0.009 | - | - | 0.004 | 0.041 | 0.065 |
|  |  | *n=156* | *n=1421* | *n=112* | *n=559* | *n=164* |  |  | *n=711* | *n=1205* | *n=417* |
| B*57:01 | 0.037 | 0.013 | 0.033 | 0.018 | 0.017 | 0.018 | 0.010 | - | 0.005 | 0.012 | 0.032 |
|  |  | *n=156* | *n=1719* | *n=112* | *n=229* | *n=363* | *n=143* |  | *n=661* | *n=859* | *n=389* |
| DRB1*07:01 | 0.156 | 0.042 | 0.149 | 0.157 | 0.043 | 0.085 | 0.031 | 0.035 | 0.031 | 0.065 | 0.086 |
|  |  | *n=83* | *n=1567* | *n=394* | *n=105* | *n=363* | *n=177* | *n=100* | *n=212* | *n=606* | *n=314* |
| DRB1*13:02 | 0.039 | - | 0.033 | 0.080 | 0.013 | 0.063 | 0.027 | - | 0.021 | 0.123 | 0.043 |
|  |  |  | *n=1567* | *n=394* | *n=80* | *n=363* | *n=94* |  | *n=212* | *n=519* | *n=314* |
| DRB1*15:01 | 0.159 | 0.042 | 0.161 | 0.072 | 0.011 | 0.067 | 0.127 | - | 0.053 | 0.030 | 0.047 |
|  |  | *n=83* | *n=1567* | *n=394* | *n=331* | *n=527* | *n=157* |  | *n=758* | *n=322* | *n=386* |
| DQA1*01:02 | NA | 0.098 | 0.219 | 0.175 | 0.031 | - | 0.472 | - | - | 0.260 | 0.161 |
|  |  | *n=107* | *n=196* | *n=183* | *n=80* |  | *n=180* |  |  | *n=406* | *n=242* |
| DQA1*02:01 | NA | 0.042 | 0.143 | 0.175 | 0.043 | - | 0.031 | 0.035 | - | 0.060 | 0.089 |
|  |  | *n=83* | *n=196* | *n=183* | *n=105* |  | *n=131* | *n=100* |  | *n=406* | *n=242* |
| DQB1*02:01 | 0.273 | 0.066 | 0.245 | 0.363 | 0.027 | - | 0.042 | 0.042 | 0.041 | 0.175 | 0.165 |
|  |  | *n=83* | *n=494* | *n=197* | *n=317* |  | *n=274* | *n=96* | *n=413* | *n=603* | *n=242* |
| DQB1*06:02 | 0.149 | 0.036 | 0.128 | 0.074 | 0.019 | - | 0.087 | - | 0.011 | 0.137 | 0.058 |
|  |  | *n=83* | *n=494* | *n=282* | *n=80* |  | *n=223* |  | *n=413* | *n=603* | *n=242* |
| DQB1*06:04 | 0.022 | - | 0.021 | 0.051 | - | - | 0.005 | - | 0.016 | 0.066 | 0.037 |
|  |  |  | *n=494* | *n=282* |  |  | *n=94* |  | *n=63* | *n=603* | *n=242* |

AUS=Australia, EUR=Europe, NAF=North Africa, NAM, North America, NEA=North East Asia, OCE=Oceania, SAM=South and Central America, SEA=South East Asia, SSA=Sub-Saharan Africa, SWA=South West Africa.

**Table s5.** **Ambiguity alleles**. The list of alleles for each allele suffix with “g”. Alleles bearing suffix 'g' in A,B,C locus have identical sequences in exon 2 and exon 3 antigen recognition sites (ARS). Alleles bearing suffix 'g' in DRB or DQB locus have identical sequences in exon 2 ARS.

| **Locus** | **ARS Allele** | **Included Alleles** | |  | |  |  |  |  |  |
| --- | --- | --- | --- | --- | --- | --- | --- | --- | --- | --- |
| A | 0101g | 01010101/01010102N/0104N/0122N | | | |  |  |  |  |  |
| A | 0201g | 02010101/02010102L/02010103/020108/020111/020114/020115/0209/0243N/0266/0275/0283N/0289/0297/9232/9234/9240 | | | | | | | | |
| A | 0206g | 020601/9226 |  | |  |  |  |  |  |  |
| A | 0207g | 0207/0215N |  | |  |  |  |  |  |  |
| A | 0211g | 0211/0269 |  | |  |  |  |  |  |  |
| A | 0301g | 03010101/03010102N/03010103/0320/0321N/0326/0337 | | | | | |  |  |  |
| A | 1101g | 110101/1121N | |  | |  |  |  |  |  |
| A | 1102 | 110201/110203 | |  | |  |  |  |  |  |
| A | 2301g | 2301/2307N/2317/2318 | |  | |  |  |  |  |  |
| A | 2402g | 24020101/24020102L/240203/240210/240213/2409N/2411N/2440N/2476/2479/2483N | | | | | | | |  |
| A | 2403g | 240301/2433 |  | |  |  |  |  |  |  |
| A | 2601g | 260101/2624/2626 | |  | |  |  |  |  |  |
| A | 2901g | 29010101/29010102N | |  | |  |  |  |  |  |
| A | 3001g | 300101/300102/3024 | |  | |  |  |  |  |  |
| A | 3002 | 300201/300202 | |  | |  |  |  |  |  |
| A | 3101g | 310102/3114N | |  | |  |  |  |  |  |
| A | 3201 | 320101/320102 | |  | |  |  |  |  |  |
| A | 3303g | 330301/3315 |  | |  |  |  |  |  |  |
| A | 6801g | 680102/6811N/6833 | |  | |  |  |  |  |  |
| A | 6802 | 68020101/68020102/68020103 | | | |  |  |  |  |  |
| B | 0702g | 070201/070206/0744/0749N/0758/0759/0761 | | | | |  |  |  |  |
| B | 0705g | 070501/0706 |  | |  |  |  |  |  |  |
| B | 0801g | 080101/0819N | |  | |  |  |  |  |  |
| B | 1501g | 15010101/15010102N/150106/150107/9502/9504/9540/9546 | | | | | |  |  |  |
| B | 1517 | 15170101/15170102 | |  | |  |  |  |  |  |
| B | 1801g | 180101/180103/1817N | |  | |  |  |  |  |  |
| B | 2705g | 270502/270504/2713 | |  | |  |  |  |  |  |
| B | 3501g | 350101/350103/3540N/3542/3557 | | | |  |  |  |  |  |
| B | 3503g | 3503/3570 |  | |  |  |  |  |  |  |
| B | 3901g | 39010101/39010102L/390103 | | | |  |  |  |  |  |
| B | 4001g | 400101/400102/4055 | |  | |  |  |  |  |  |
| B | 4002g | 400201/4056 |  | |  |  |  |  |  |  |
| B | 4006 | 40060101/40060102 | |  | |  |  |  |  |  |
| B | 4402g | 44020101/44020102S/4419N/4427 | | | |  |  |  |  |  |
| B | 4403 | 440301/440303 | |  | |  |  |  |  |  |
| B | 4501g | 4501/4507 |  | |  |  |  |  |  |  |
| B | 4701 | 47010101/47010102 | |  | |  |  |  |  |  |
| B | 5101g | 510101/510105/510107/5111N/5130/5132/5148/5151 | | | | |  |  |  |  |
| B | 5201g | 520101/5207 |  | |  |  |  |  |  |  |
| B | 5501 | 550101/550103 | |  | |  |  |  |  |  |
| B | 5801g | 580101/5811 |  | |  |  |  |  |  |  |
| B | 8101g | 8101/8102/8103 | |  | |  |  |  |  |  |
| C | 0102 | 010201/010202 | |  | |  |  |  |  |  |
| C | 0302 | 030201/030202/030203 | |  | |  |  |  |  |  |
| C | 0303g | 030301/0320N | |  | |  |  |  |  |  |
| C | 0304 | 030401/030403 | |  | |  |  |  |  |  |
| C | 0401g | 04010101/04010102/0409N/0428/0430 | | | |  |  |  |  |  |
| C | 0501g | 050101/050104/0503 | |  | |  |  |  |  |  |
| C | 0602 | 06020101/06020102/060203 | | | |  |  |  |  |  |
| C | 0701g | 070101/070102/070109/0706/0718/0752 | | | |  |  |  |  |  |
| C | 0702g | 07020101/07020102/07020103/0750 | | | |  |  |  |  |  |
| C | 0704g | 070401/0711 |  | |  |  |  |  |  |  |
| C | 1202 | 120201/120202 | |  | |  |  |  |  |  |
| C | 1203 | 12030101/12030102/120306 | | | |  |  |  |  |  |
| C | 1502g | 150201/1513 |  | |  |  |  |  |  |  |
| C | 1505 | 150501/150502/150503 | |  | |  |  |  |  |  |
| C | 1801g | 1801/1802 |  | |  |  |  |  |  |  |
| DQB1 | 0201g | 020101/0202/0204 | |  | |  |  |  |  |  |
| DQB1 | 0301g | 030101/030104/0309/0319/0321 | | | |  |  |  |  |  |
| DQB1 | 0601 | 060101/060103 | |  | |  |  |  |  |  |
| DQB1 | 0604g | 060401/0634 |  | |  |  |  |  |  |  |
| DRB1 | 0801 | 080101/080103 | |  | |  |  |  |  |  |
| DRB1 | 1201g | 120101/1206/1210 | |  | |  |  |  |  |  |
| DRB1 | 1401g | 140101/1454 |  | |  |  |  |  |  |  |

Reference - Human Immunology 68, 392-417 (2007) @American Society for Histocompatibility and Immunogenetics, 2007

**Table s6.** **LD for alleles represented in Figure 2.** Counts (frequencies) of Caucasian individuals from the ENW archive (n=298) who carry alleles and two loci haplotypes implicated in DILI and percentage of allele carriers who also carry the haplotypes are shown here.

| **Allele** | **Count (Frequency)** | **Haplotype** | **Count (Frequency)** | **Percentage of allele carriers who carry the haplotype** |
| --- | --- | --- | --- | --- |
| B*5701 | 21 (0.07) | B*5701, DRB1*0701 | 19 (0.064) | 90% |
| DQB1*0303 | 29 (0.097) | DQB1*0303, DRB1*0701 | 22 (0.074) | 76% |
| DRB1*0701 | 86 (0.289) | DQB1*0201, DRB1*0701 | 69 (0.23) | 80% |
| B*0801 | 89 (0.299) | B*0801, DQB1*0201 | 74 (0.25) | 83% |
| DQB1*0602 | 82 (0.275) | DQB1*0602, DRB1*1501 | 81 (0.272) | 99% |
| DQB1*0604 | 13 (0.044) | DRB1*1302, DQB1*0604 | 13 (0.044) | 100% |
| A*3303 | 2 (0.007) | A*3303, DRB1*1302 | 1 (0.0036) | 50% |
| DQB1*0602 | 82 (0.275) | DQB1*0602, DRB1*0701 | 13 (0.043) | 15.90% |

**Figure s1. Frequency distribution of HLA alleles associated with DILI.**

Alleles associated with DILI were selected from the Allele Frequency Net Database and their frequencies plotted. It is interesting to note higher frequency for the HLA-A*33:03 allele in Japanese compared with Caucasians. A*33:03 has been implicated in ticlopidine-induced hepatotoxicity in Japanese population. Colour of the dots indicate HLA-allele frequency (blue from 0 to 0.099; green from 0.10 to 0.249; red from 0.25 to 1)


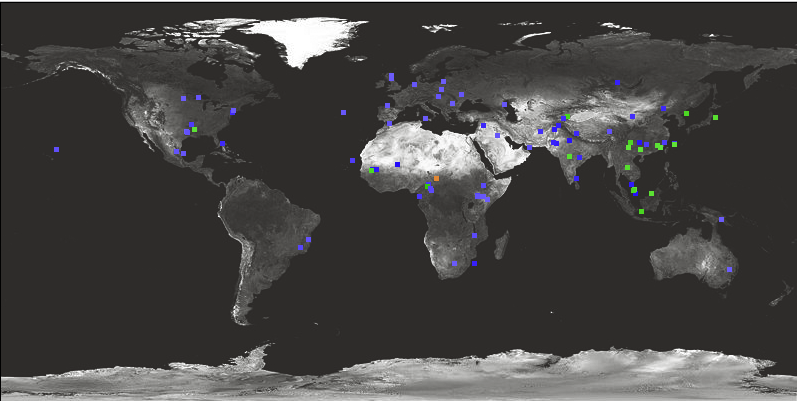

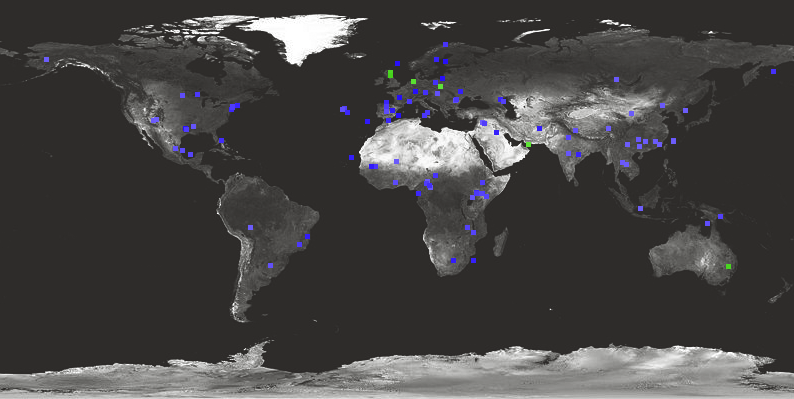


**A*33:03**

**B*08:01**


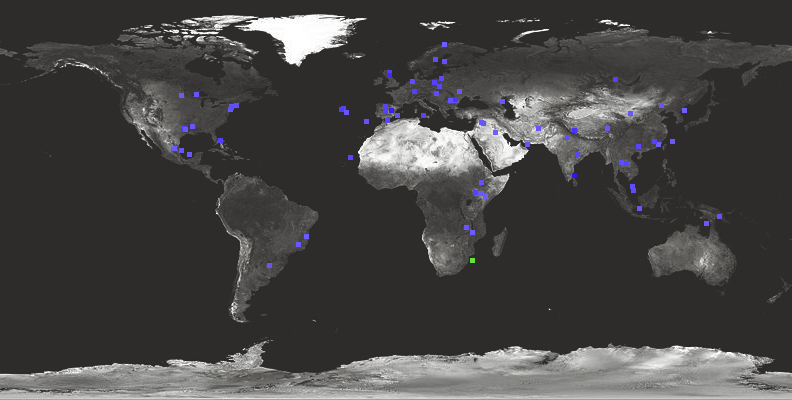

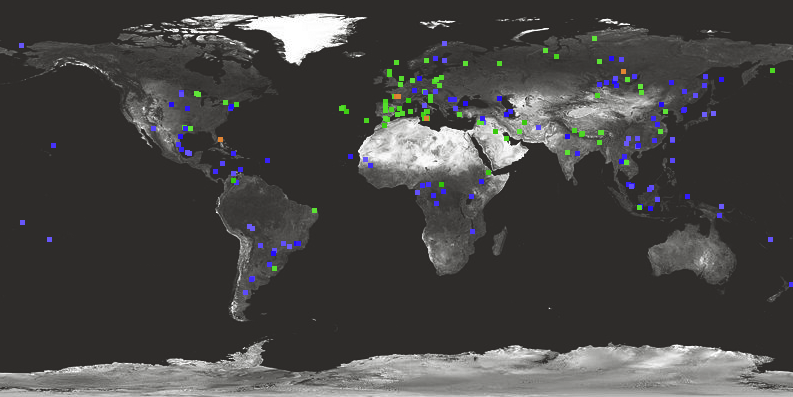


**B*57:01**

**DRB1*07:01**


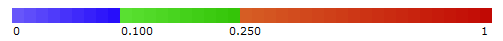


**Supplementary Figure 1 (Cont)**

**
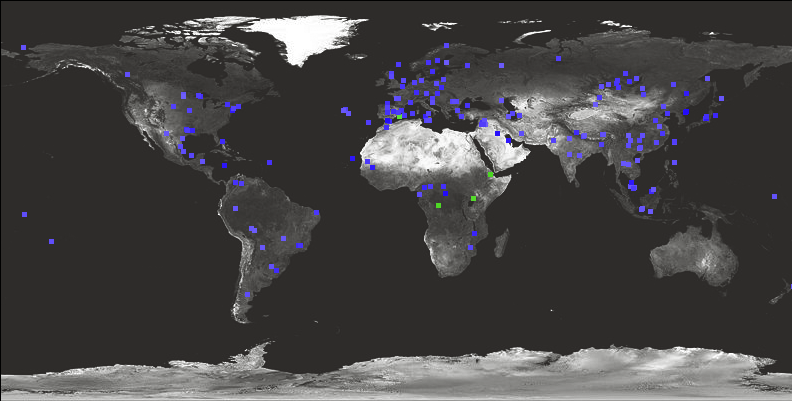
**


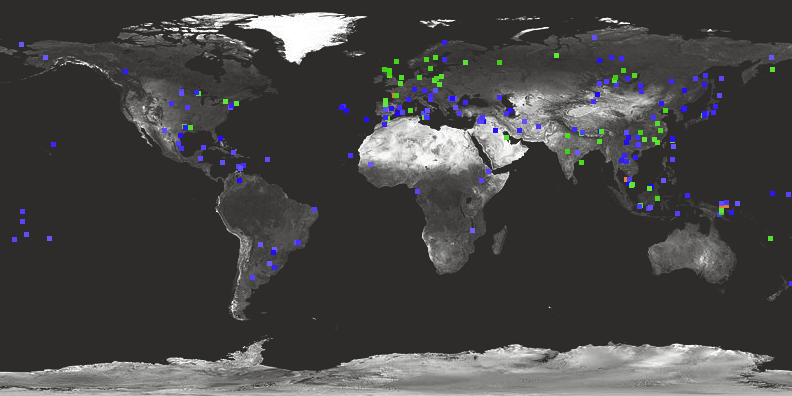


**DRB1*13:02**

**DRB1*15:01**


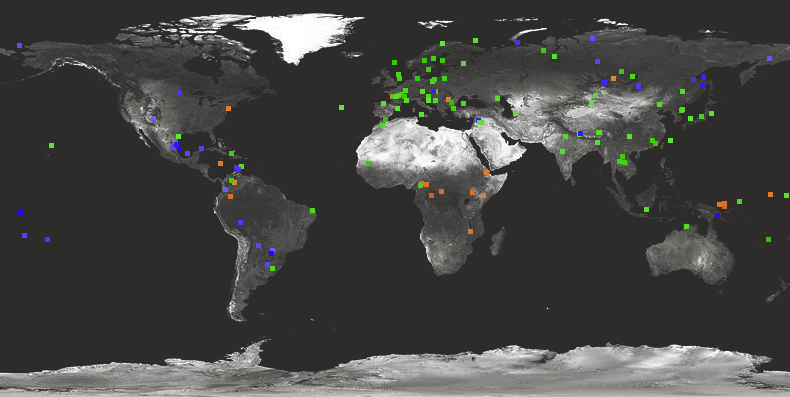

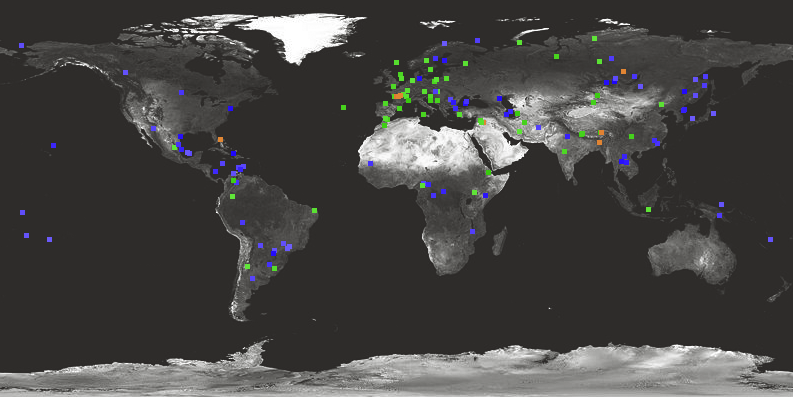


**DQA1*01:02**

**DQA1*02:01**

**Supplementary Figure 1 (Cont)**


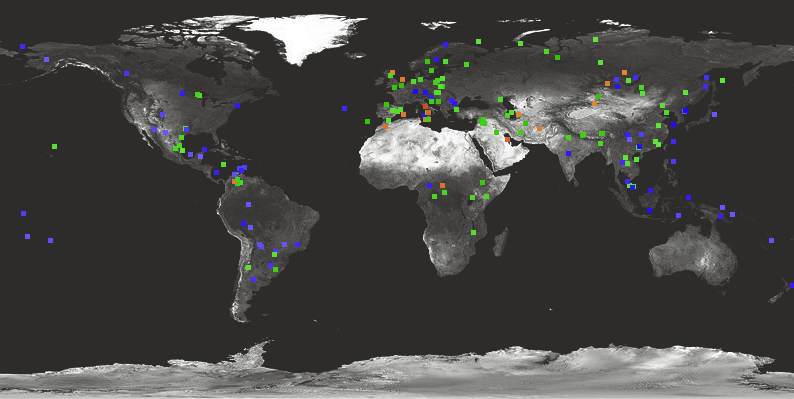

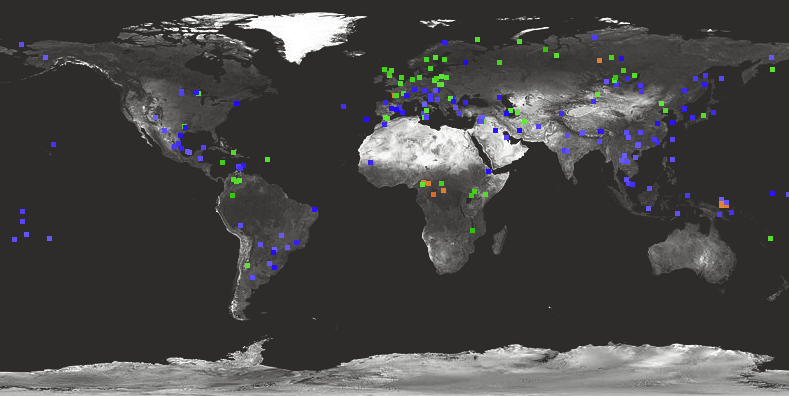


**DQB1*02:01**

**DQB1*06:02**


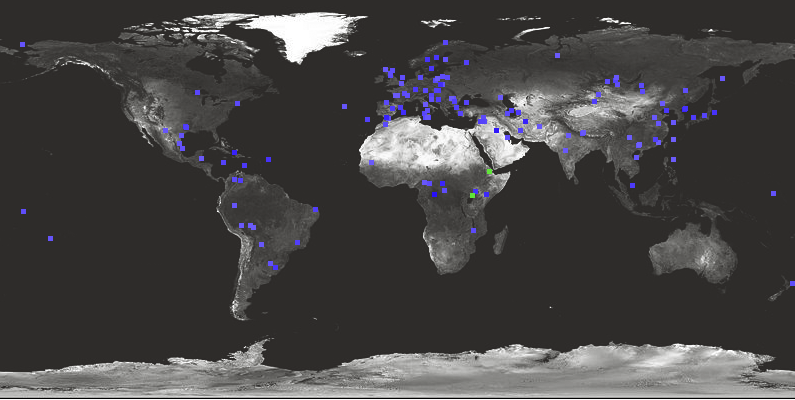


**DQB1*06:04**
